# Supplementary material for: The impact of health care on outcomes of suspected testicular torsion: results from the GRAND study
Source: World J Urol. 2024 May 9;42(1):309. doi: 10.1007/s00345-024-05015-z (PMC11082016; doi:10.1007/s00345-024-05015-z)
Supplement: Supplementary file 2 — Supplementary file2 (DOCX 76 KB) [file 345_2024_5015_MOESM2_ESM.docx]

**Title page**

**The impact of health care on outcomes of suspected testicular torsion: Results from the GRAND study**

Nikolaos Pyrgidis^1^, Maria Apfelbeck^1^, Regina Stredele^1^, Severin Rodler^1^, Marc Kidess^1^, Yannic Volz^1^, Philipp Weinhold^1^, Christian G. Stief^1^, Julian Marcon^1^, Gerald B. Schulz^1^, Michael Chaloupka^1^

**Authors’ affiliation:**

1. Department of Urology, University Hospital, LMU Munich, Germany.

**Corresponding author**: Nikolaos Pyrgidis

Address: Marchioninistraße 15, 81377 Munich

Telephone number: 0049 152 5484 7591

Fax: +49-89-4400-78890

Email: [Nikolaos.pyrgidis@med.uni-muenchen.de](mailto:Nikolaos.pyrgidis@med.uni-muenchen.de)

**Running title:** Testicular torsion and perioperative outcomes

**Manuscript word count:** 2636

**Abstract word count:** 295

**Figures:** 1

**Tables:** 3

# Abstract

**Background:** Suspicion of testicular torsion represents a urological emergency, necessitating immediate surgery. Comprehensive data on the current trends and perioperative outcomes regarding surgical exploration are sparse. Therefore, we utilized nationwide data on the prevalence and results of this surgery, aiming to provide evidence on this matter.

**Methods:** We assessed the GeRmAn Nationwide inpatient Data (GRAND) from 2005 to 2021, provided by the Research Data Center of the Federal Bureau of Statistics. We performed multiple regression analyses to evaluate the perioperative outcomes (length of hospital stay, transfusion, and surgical wound infection) after surgical exploration due to suspected testicular torsion based on both the outcome of surgery (orchiectomy, detorsion with preservation of the testicle, and no testicular torsion) and on the department of operation (urological versus non-urological).

**Results:** A total of 81,899 males underwent surgical exploration due to suspected testicular torsion in Germany from 2005 to 2021. Of them, 11,725 (14%) underwent orchiectomy, 30,765 (38%) detorsion with preservation of the testicle and subsequent orchidopexy, and 39,409 (48%) presented no testicular torsion. Orchiectomy was significantly associated with longer length of hospital stay (day difference of 1.4 days, 95%CI: 1.3-1.4, p<0.001), higher odds of transfusion (1.8, 95% CI: 1.2-2.6, p=0.002) and surgical wound infections (1.8, 95%CI: 1.4-2.3, p<0.001) compared to no testicular torsion. The proportion of patients undergoing orchiectomy was significantly lower in urological departments (14%) versus non-urological departments (16%) and the proportion of patients undergoing preservation of testicle after detorsion was significantly higher in urological departments (38%) versus non-urological departments (37%), p<0.001. Patients undergoing treatment in a urological department were discharged earlier and presented lower odds of transfusion and surgical wound infection (p<0.001) compared to patients undergoing treatment in a non-urological department.

**Conclusions:** Nearly half of patients who underwent surgery for suspected testicular torsion did not have intraoperatively the condition confirmed. Patients treated in urological departments had significantly better perioperative outcomes compared to those treated in non-urological departments. Therefore, we advise to refer patients to urological treatment as early as possible.

**Keywords:** Testicular Torsion, Urologic emergency, Orchiectomy, Perioperative Outcomes, Cohort Study

**Introduction**

Suspected testicular torsion is an absolute urological emergency manifesting with acute and severe pain and merits immediate surgical exploration ^1^. Administrative data suggest that, among patients undergoing surgical exploration for suspected testicular torsion, preservation of the testicle after detorsion and subsequent orchidopexy is achieved in approximately 36% of the cases, orchiectomy due to testicular necrosis is mandatory in 31% of the cases, while no testicular torsion is identified in about 33% of the cases ^2^. Among patients with no testicular torsion, a significant number present with torsion of the appendix testis, epididymitis, inguinal hernia, or other causes of acute testicular pain ^3^.

The diagnosis of acute testicular torsion is based primarily on clinical signs and symptoms, although Doppler ultrasound might be useful in some cases ^4^. Doppler ultrasound has a sensitivity of approximately 100%, a specificity of 80%, a positive predictive value of 82% and a negative predictive value of 100% ^5^. However, it should be highlighted that a testicular torsion can be only safely excluded after surgical exploration ^6^. The incidence of testicular torsion is about one case per 4,000 males per year in those younger than 25 years old and one case per 160 males over a lifetime in men older than 25 years ^7^. The testicular salvage rate is approximately 90% if the detorsion of the spermatic cord is performed within 6 hours from the onset of symptoms, whereas this salvage rate falls to 50% after 12 hours and below 10% after 24 hours ^8^.

Even though testicular torsion is a urologic emergency, a significant number of patients are not treated in a urology department. Other non-urologic departments such as pediatric or general surgery may also operate patients with suspected testicular torsion ^9^. Given that urologists present greater experience with surgery of the external male genitalia, treatment on a urology department may present better perioperative outcomes. However, to date, no comprehensive epidemiological studies on this matter exist ^10^. Accordingly, studies exploring the current trends and perioperative outcomes after surgical exploration due to suspected testicular torsion are sparse. In this context, we aimed to provide nationwide evidence on the current trends, risk factors, and important perioperative outcomes of men undergoing surgical exploration for suspected testicular torsion through the largest study on the field.

**Methods**

**GeRmAn Nationwide inpatient Data (GRAND)**

The GRAND study contains nationwide in-hospital data in Germany from 2005 to 2021 except for psychiatric, forensic, and military cases. These data are stored anonymized at the Research Data Center of the Federal Bureau of Statistics and were retrieved for the purposes of this analysis after agreement (LMU - 4710-2022). Our research team did not have direct access to these data on a patient level but could only access their summary results. In particular, all analyses were performed, on our behalf, from the Research Data Center based on R codes developed by our research team. Subsequently, the summary results were provided by the Research Data Center of the Federal Bureau of Statistics to our research group for further evaluation (source: Research Data Center of the Federal Bureau of Statistics, DRG Statistics 2005-2021, own calculations).

Since 2005, all hospitals in Germany are required to transfer all in-hospital data to the German Institute for the Hospital Remuneration System to receive their remuneration. These data are coded according to the International Statistical Classification of Diseases and Related Health Problems, 10^th^ revision, German modification (ICD-10-GM), and the German Procedure Classification (OPS) and contain information on baseline diagnoses, in-hospital procedures, and perioperative outcomes. Following the German legislation, approval by an ethics committee or patient informed consent was not mandatory for the present analysis from the GRAND study.

**Outcomes**

We included all hospitalized cases with suspicion of testicular torsion (ICD-10-GM: N44.0 and N44.1) in Germany. The primary outcome of the study was to assess the current trends in the management of suspected testicular torsion. Secondary outcomes included perioperative complications (length of hospital stay, transfusion, and surgical wound infection) in patients undergoing orchiectomy, detorsion with preservation of the testicle and subsequently orchidopexy, and in those with no testicular torsion. Moreover, we assessed the perioperative outcomes (type of surgery, length of hospital stay, transfusion, and surgical wound infection) based on the department of operation (urological versus non-urological).

**Statistical analysis**

All categorical variables were summarized as frequencies with proportions and were compared with the chi-squared test. Accordingly, all continuous variables were reported as median with interquartile range (IQR) and were compared with the Kruskal-Wallis test. We performed a multivariable logistic and linear regression analysis to assess the perioperative outcomes (length of hospital stay, transfusion, and surgical wound infection) after surgical exploration due to suspected testicular torsion based on both the outcome of surgery (orchiectomy, detorsion with preservation of the testicle, and no testicular torsion) and on the department of operation (urological versus non-urological). Based on clinical relevance, all models were adjusted for age, obesity, and diabetes. The odds ratios (ORs) with the 95% confidence intervals (CIs) were reported for all logistic models and two-sided p-values <0.05 were considered statistically significant.

**Results**

**Baseline characteristics**

A total of 81,899 males with a median age of 16 (IQR: 13, 23) years have undergone surgical exploration due to suspected testicular torsion in Germany from 2005 to 2021. Of them, 11,725 (14%) underwent an orchiectomy, 30,765 (38%) detorsion with preservation of the testicle and subsequent orchidopexy, and 39,409 (48%) presented no testicular torsion. Among patients with no testicular torsion, torsion of the appendix testis was the cause of acute testicular pain in 9,861 (25%) cases and epididymitis in 3,161 (8%) cases. Compared to patients with intraoperative detorsion of the testicle and no testicular torsion, patients undergoing orchiectomy presented higher rates of diabetes, chronic kidney disease, hypertension, and obesity (p < 0.001 for all characteristics). Interestingly, a higher proportion of males undergoing orchiectomy had a history of cryptorchidism (p < 0.001). Furthermore, the hospital revenues were higher after orchiectomy (p < 0.001). The baseline characteristics of all included males are depicted in Table 1.

The total number of patients undergoing surgical exploration due to suspected testicular torsion in Germany has decreased in the last years from 5,235 cases in 2005 to 4,900 cases in 2021 (p < 0.001). The latter is attributed to the decrease in the cases of surgical exploration with no testicular torsion which decreased from 2,688 in 2005 to 2,248 in 2021. On the other hand, both the number of orchiectomies and the number of preservations of testicle after detorsion has slightly increased (from 733 and 1,814 in 2005 to 773 and 1,879 in 2021, accordingly). The annual trends of orchiectomy, detorsion with preservation of the testicle, and no testicular torsion are presented in Figure 1.

**Perioperative outcomes of suspected testicular torsion**

The median length of hospital stay was 1 day (IQR: 1, 2) for patients with no testicular torsion, 1 day (IQR: 1, 2) for those with detorsion with preservation of testicle, and 2 days (IQR: 2, 4) for those undergoing orchiectomy. The rates of transfusions and surgical wound infections were low in all groups. In the multivariate analysis after adjusting for age, obesity, and diabetes, orchiectomy was significantly associated with longer length of hospital stay (2 versus 1 days, day difference of 1.4 days, 95% CI: 1.3 to 1.4, p < 0.001) compared to no testicular torsion, whereas preservation of testicle after detorsion was associated with shorter length of hospital stay (day difference of 0.08 days, 95% CI: 0.02 to 0.14, p < 0.001) compared to no testicular torsion. Similarly, orchiectomy was associated with significantly higher odds of transfusion (0.4% versus 0.2%, OR: 1.8, 95% CI: 1.2 to 2.6, p = 0.002), and preservation of testicle after detorsion was associated with significantly lower odds of transfusion (<0.1% versus 0.2%, OR: 0.27, 95% CI: 0.15 to 0.47, p < 0.001) compared to no testicular torsion. Accordingly, orchiectomy was associated with significantly higher odds of surgical wound infections (0.9% versus 0.5%, OR: 1.8, 95% CI: 1.4 to 2.3, p < 0.001), and preservation of testicle after detorsion was associated with significantly lower odds of surgical wound infections (0.3% versus 0.5%, OR: 0.67, 95% CI: 0.52 to 0.85, p < 0.001) compared to no testicular torsion. All regression models are available in Table 2.

**Perioperative outcomes based on the department of treatment**

A total of 61,491 (75%) surgical explorations due to suspected testicular torsion were undertaken in a urological department and 20,408 (25%) in a non-urological department. Among them, the proportion of patients undergoing orchiectomy was significantly lower in urological departments (8,383, 14%) versus non-urological departments (3,342, 16%) and the proportion of patients undergoing preservation of testicle after detorsion and subsequent orchidopexy was significantly higher in urological departments (23,238, 38%) versus non-urological departments (7,527, 37%), p < 0.001.

In the multivariate analysis, patients undergoing treatment in a urological department were discharged 1.4 days (95% CI: 1.3 to 1.4, p < 0.001) earlier compared to patients undergoing treatment in a non-urological department. Accordingly, treatment in a urological department was associated with significantly lower odds of transfusion (0.05, 95% CI: 0.03 to 0.08, p < 0.001) and surgical wound infection (0., 95% CI: 1.4 to 2.3, p < 0.001) compared to a non-urological department. All regression models are displayed in Table 3.

# **Discussion**

The results of the present GRAND analysis show that almost half of all men undergoing surgical exploration for suspected testicular torsion do not present a testicular torsion intraoperatively. Even though this number has slightly decreased in the last years, it remains alarmingly high. Of note, the total number of perioperative complications in men with suspected testicular torsion is relatively low. However, as expected, patients undergoing orchiectomy have the highest rates of perioperative complications, and men with no testicular torsion have more perioperative complications than males with detorsion with preservation of testicular torsion. The latter may be explained by the fact that patients with testicular pain, but no testicular torsion, may present other conditions with higher rates of perioperative complications such as epididymitis or inguinal hernia. It should be also highlighted that our findings suggest that patients undergoing surgery in a urological department presented better perioperative outcomes and higher rates of testicular preservation in cases of testicular torsion.

It seems that testicular torsion occurs in Germany at a higher age compared to other countries. Nationwide data from the USA, Korea, and Ireland indicate that the commonest age for testicular torsion is 10 to 14 years ^2,11,12^. On the other hand, the ratio of orchiectomy to preservation of testicle after detorsion in Germany is 1:3, which is similar to other Western countries such as the USA and Ireland ^11,13^. Importantly, the decreasing trends in patients with no testicular torsion who undergo surgical exploration may be attributed to the improvement of imaging modalities used for the assessment of patients with suspected testicular torsion ^14,15^. Moreover, our analysis demonstrates that cryptorchidism is a risk factor for testicular torsion in males that have not previously undergone orchidopexy. The latter has been recognized by previous studies suggesting that testicular torsion in these patients should be suspected, diagnosed, and managed without delay ^16^.

Overall, the number of perioperative complications in patients with suspected testicular torsion seems to be relatively low in Germany and is comparable to other Western countries. Based on our findings, the length of hospital stay was short, patients rarely required transfusion, and the rates of surgical wound infections were below 1%. These findings are in line with previous studies focusing on perioperative complications. In particular, administrative data from the USA indicate that the length of hospital stay after surgical exploration for suspected testicular torsion is about one day, transfusions are rare, and surgical wound infections occur in about 1% of all cases. Accordingly, severe complications such as admission to an intensive care unit, myocardial infarction, pulmonary embolism, sepsis, or acute renal disease occur in extremely rare cases and, therefore, it was beyond the scope of the present analysis to assess their incidence ^13^.

Emergency care for suspected testicular torsion is generally provided by urology, pediatric surgery, and general surgery. To our knowledge, our study is the first to compare nationwide data on outcomes of this surgery between different departments. Our findings indicate that patients should preferably undergo surgical exploration in urological departments. In particular, the hospital stay was shorter by 1.4 days, the transfusion rates were lower by 5% and the surgical wound infections were lower by 40%. Importantly, the orchiectomy rates were 2% lower in patients undergoing surgical exploration of suspected testicular torsion in a urological department versus a non-urological department. Nevertheless, it should be highlighted that only 75% of all patients with suspected testicular torsion undergo surgical exploration in a urological department. The latter might be attributed to the fact that patients younger than 18 are primarily assessed by pediatric surgeons and by the fact that about 50% of all hospitals in Germany do not have a urological department.

Our findings indicate that a correct and timely diagnosis of testicular torsion is mandatory to save the testicle and prevent unnecessary surgeries. Indeed, our epidemiological study on the German population suggests that an important number of patients, who require surgical exploration for suspected testicular torsion, do not present a testicular torsion intraoperatively. Moreover, although our study shows better perioperative outcomes in hands of urologists, it should be highlighted that testicular torsion is an absolute emergency ^17^. Therefore, general, and pediatric surgeons should also be able to perform it when necessary. It seems that a better distribution and availability of urology departments throughout the country is mandatory ^18^. Accordingly, a urologist-guided training of non-urological surgical fellows for the initial evaluation of acute scrotum might lead to better outcomes ^19^.

Even though we present, to our knowledge, the largest analysis of trends in the surgical management of suspected testicular torsion, our findings were mitigated by some limitations. The GRAND study is based on retrospective, administrative data, which are prone to coding errors, although they are checked by multiple independent task forces. It should be noted that important baseline and perioperative data such as patients’ laboratory findings, previous attempts of manual detorsion, duration of testicular torsion, rates of orchidopexy for the contralateral side, operative time, postoperative care, as well as long-term complications, readmission, and reoperation rates are not included in the GRAND database. Moreover, no information on the use of Doppler prior to surgical exploration of suspected testicular torsion is available and the preoperative clinical findings could not be correlated with the intraoperative findings. The lack of these data introduces a certain selection bias to the present findings. Based on the previous notion, the observed better outcomes in urology departments compared to non-urology departments might be attributed to the inherent differences in the baseline characteristics of the included patients (e.g. less comorbidities, prompt presentation to the urology department), for which we could not account. It was also beyond the scope of the present analysis to identify all causes of testicular pain imitating acute testicular torsion. Importantly, all our findings derive from nationwide German data and, thus, they might not be extrapolated to other medical healthcare systems. Nevertheless, in an attempt to overcome these limitations, our holistic approach with multiple high-volume analyses may serve predominantly as a valuable guide for proper patient counseling and for the referral of patients with suspected testicular torsion to urological departments, whenever possible.

# **Conclusion**

The present real-world data indicate that an important amount of patients with suspected testicular torsion undergoing surgery do not in fact present a testicular torsion. Even though the proportion of males with no testicular torsion undergoing surgery has decreased, the proportion of patients undergoing unnecessary surgery is high. Overall, the present analysis suggests that surgical exploration of suspected testicular torsion should be preferably performed in urological departments due to better perioperative outcomes and, importantly, due to lower rates of orchiectomy and higher rates of saving of the testicle. Nevertheless, it should be stressed that, due to the selection bias of the present cohort and the limitations of the analysis, no definite conclusions can be drawn and further studies in the field are mandatory.

**Acknowledgments:** None.

**Author contributions**: All authors participated in the drafting, writing, and editing of the manuscript.

**Ethics statement:** Written informed consent from the participants, as well as ethical approval, was not required for the present study in accordance with the national legislation and institutional requirements. All data used in this work are stored anonymized at the German Federal Statistical Office.

**Conflict of interest:** None declared.

**Funding information:** This research did not receive any specific grant from funding agencies in the public, commercial, or not-for-profit sectors.

# References

1. Somani BK, Watson G, Townell N. Testicular torsion. *BMJ*. 2010;341:c3213. doi:10.1136/bmj.c3213

2. Zhao LC, Lautz TB, Meeks JJ, Maizels M. Pediatric testicular torsion epidemiology using a national database: incidence, risk of orchiectomy and possible measures toward improving the quality of care. *J Urol*. 2011;186(5):2009-2013. doi:10.1016/j.juro.2011.07.024

3. Rakha E, Puls F, Saidul I, Furness P. Torsion of the testicular appendix: importance of associated acute inflammation. *J Clin Pathol*. 2006;59(8):831-834. doi:10.1136/jcp.2005.034603

4. Mori T, Ihara T, Nomura O. Diagnostic accuracy of point-of-care ultrasound for paediatric testicular torsion: a systematic review and meta-analysis. *Emerg Med J*. 2023;40(2):140-146. doi:10.1136/emermed-2021-212281

5. Qin KR, Qu LG. Diagnosing with a TWIST: Systematic Review and Meta-Analysis of a Testicular Torsion Risk Score. *J Urol*. 2022;208(1):62-70. doi:10.1097/JU.0000000000002496

6. Altinkilic B, Pilatz A, Weidner W. Detection of normal intratesticular perfusion using color coded duplex sonography obviates need for scrotal exploration in patients with suspected testicular torsion. *J Urol*. 2013;189(5):1853-1858. doi:10.1016/j.juro.2012.11.166

7. Barada JH, Weingarten JL, Cromie WJ. Testicular salvage and age-related delay in the presentation of testicular torsion. *J Urol*. 1989;142(3):746-748. doi:10.1016/s0022-5347(17)38875-4

8. Karaguzel E, Kadihasanoglu M, Kutlu O. Mechanisms of testicular torsion and potential protective agents. *Nat Rev Urol*. 2014;11(7):391-399. doi:10.1038/nrurol.2014.135

9. Chun B, Colaco M, Fox JA, et al. Salvage Surgery Rates for Pediatric Testicular Torsion: Comparison of the Pre- and Post-Quality Metric Eras. *J Urol*. 2022;208(2):441-447. doi:10.1097/JU.0000000000002677

10. Chiu B, Chen CS, Keller JJ, Lin CC, Lin HC. Seasonality of testicular torsion: a 10-year nationwide population based study. *J Urol*. 2012;187(5):1781-1785. doi:10.1016/j.juro.2011.12.104

11. Sugrue DD, O’Connor E, Davis N. Testicular torsion in Ireland: a 10-year analysis of incidence and risk of orchidectomy. *Ir J Med Sci*. 2022;191(5):2267-2274. doi:10.1007/s11845-021-02816-8

12. Choi JB, Han KH, Lee Y, et al. The incidence of testicular torsion and testicular salvage rate in Korea over 10 years: A nationwide population-based study. *Investig Clin Urol*. 2022;63(4):448-454. doi:10.4111/icu.20220122

13. Brungardt JG, McLeay MT, Schropp KP. Testicular torsion in adults: Demographics and 30-day outcomes after orchiopexy or orchiectomy. *Curr Urol*. 2021;15(4):219-224. doi:10.1097/CU9.0000000000000032

14. Samson P, Hartman C, Palmerola R, et al. Ultrasonographic Assessment of Testicular Viability Using Heterogeneity Levels in Torsed Testicles. *J Urol*. 2017;197(3 Pt 2):925-930. doi:10.1016/j.juro.2016.09.112

15. Clement KD, Light A, Asif A, et al. A BURST-BAUS consensus document for best practice in the conduct of scrotal exploration for suspected testicular torsion: the Finding consensus for orchIdopeXy In Torsion (FIX-IT) study. *BJU Int*. 2022;130(5):662-670. doi:10.1111/bju.15818

16. Pogorelić Z, Mrklić I, Jurić I, Biočić M, Furlan D. Testicular torsion in the inguinal canal in children. *J Pediatr Urol*. 2013;9(6 Pt A):793-797. doi:10.1016/j.jpurol.2012.10.013

17. Seizilles de Mazancourt E, Khene Z, Sbizerra M, et al. Cut-off time for surgery and prediction of orchiectomy in spermatic cord torsion: a retrospective multicentric study over 15 years. *World J Urol*. 2023;41(12):3789-3794. doi:10.1007/s00345-023-04671-x

18. Friedman AA, Ahmed H, Gitlin JS, Palmer LS. Standardized education and parental awareness are lacking for testicular torsion. *J Pediatr Urol*. 2016;12(3):166.e1-8. doi:10.1016/j.jpurol.2016.01.008

19. Yap LC, Keenan R, Khan J, et al. Parental awareness of testicular torsion amongst Irish parents. *World J Urol*. 2018;36(9):1485-1488. doi:10.1007/s00345-018-2269-8

| **Characteristic** | **Overall**, n = 81,899 | **Orchiectomy**, n = 11,725 | **Detorsion with preservation of testicle**, n = 30,765 | **No testicular torsion**, n = 39,409 | **p-value** |
| --- | --- | --- | --- | --- | --- |
| **Age (years)** | 16 (13-23) | 16 (13-27) | 16 (13-21) | 17 (13-23) | **<0.001** |
| **Hospital revenues (Euros)** | 1,962 (1,735-2,317) | 2,409 (2,036-3,014) | 1,954 (1,755-2,304) | 1,859 (1,630-2,225) | **<0.001** |
| **Diabetes** | 485 (0.6%) | 141 (1.2%) | 126 (0.4%) | 218 (0.6%) | **<0.001** |
| **Chronic kidney disease** | 175 (0.2%) | 72 (0.6%) | 23 (<0.1%) | 80 (0.2%) | **<0.001** |
| **Hypertension** | 1,265 (1.5%) | 442 (3.8%) | 247 (0.8%) | 576 (1.5%) | **<0.001** |
| **Obesity** | 843 (1%) | 160 (1.4%) | 248 (0.8%) | 435 (1.1%) | **<0.001** |
| **Hypospadias** | 64 (<0.1%) | 14 (0.1%) | 16 (<0.1%) | 34 (<0.1%) | 0,061 |
| **Cryptorchidism** | 1,047 (1.3%) | 323 (2.8%) | 246 (0.8%) | 478 (1.2%) | **<0.001** |
| **Department** |  |  |  |  | **<0.001** |
| Non-urological | 20,408 (25%) | 3,342 (29%) | 7,527 (24%) | 9,539 (24%) |  |
| Urological | 61,491 (75%) | 8,383 (71%) | 23,238 (76%) | 29,870 (76%) |  |

Table 1: Baseline characteristics of patients with suspected testicular torsion. Variables are presented as median with interquartile range or frequencies with proportions. The Kruskal-Wallis test was performed for comparisons among continuous variables and the chi-squared test for categorical variables. The bold cells indicate statistically significant p-values.

|  | **Hospital stay** | | | **Transfusion** | | | **Surgical wound infection** | | |
| --- | --- | --- | --- | --- | --- | --- | --- | --- | --- |
| **Outcome of surgical exploration** | **Days** | **Day difference** | **p-value** | **Events** | **OR** | **p-value** | **Events** | **OR** | **p-value** |
| No testicular torsion | 1 (1, 2) | — | — | 73 (0.2%) | — | — | 188 (0.5%) | — | — |
| Detorsion with preservation of testicle | 1 (1, 2) | -0.08 (-0.14, -0.02) | **<0.011** | 14 (<0.1%) | 0.27 (0.15, 0.47) | **<0.001** | 96 (0.3%) | 0.67 (0.52, 0.85) | **<0.001** |
| Orchiectomy | 2 (2, 4) | 1.4 (1.3, 1.4) | **<0.001** | 52 (0.4%) | 1.8 (1.2, 2.6) | **0.002** | 110 (0.9%) | 1.8 (1.4, 2.3) | **<0.001** |

Table 2: Multivariable logistic and linear regression analysis comparing length of hospital stay, transfusion rates and surgical wound infection rates after surgical exploration due to suspected testicular torsion. All models are adjusted for age, obesity, and diabetes. The bold cells indicate statistically significant p-values. OR: odds ratio.

|  | **Hospital stay** | | **Transfusion** | | **Surgical wound infection** | |
| --- | --- | --- | --- | --- | --- | --- |
| **Department** | **Day difference** | **p-value** | **OR** | **p-value** | **OR** | **p-value** |
| Non-urological | — | — | — | — |  |  |
| Urological | -1.4 (-1.4, -1.3) | **<0.001** | 0.05 (0.03, 0.08) | **<0.001** | 0.4 (0.33, 0.5) | **<0.001** |

Table 3: Multivariable logistic and linear regression analysis comparing length of hospital stay, hospital revenues, transfusion rates and surgical wound infection rates based on the department of operation. All models are adjusted for age, obesity, and diabetes. The bold cells indicate statistically significant p-values. OR: odds ratio.

# Figures

#

Figure 1: The annual number of cases with suspected testicular torsion.
