# Supplementary material for: The impact of health care on outcomes of suspected testicular torsion: results from the GRAND study
Source: World J Urol. 2024 May 9;42(1):309. doi: 10.1007/s00345-024-05015-z (PMC11082016; doi:10.1007/s00345-024-05015-z)
Supplement: Supplementary file 1 — Supplementary file1 (DOCX 56 KB) [file 345_2024_5015_MOESM1_ESM.docx]

| Klinikum der Universität München · Urologische Klinik und Poliklinik · | |  | Telefon +49 (0)89 4400 - 0  Telefax +49 (0)89 4400- 78890  http://uro.klinikum.uni-muenchen.de  Postanschrift:  Marchioninistr. 15  D-81377 München  Öffentl. Verkehr: U6, 56, 266, 269 oder N41 bis Haltestelle Klinikum Großhadern |
| --- | --- | --- | --- |
| *Editor-in-Chief*  World Journal of Urology Editor Office | |  |  |
|  |  |  | Munich, March 5th 2024 |

Dear Dr. de la Taille,

Thank you for giving us the opportunity to resubmit our cohort study to the World Journal of Urology. We appreciate the thoughtful reviews and thought-provoking suggestions provided by Reviewer 2, and 4. We believe that their comments serve to strengthen our article. Therefore, we have included all their suggestions in our manuscript, and we have addressed each comment in a point-by-point response below. All changes in the manuscript are highlighted. Accordingly, a “clear” version of our manuscript is also provided. The revised manuscript has been developed in consultation with all co-authors and each author has approved the final form of the manuscript.

**Reviewer #2**

***This is a good epidemiological study done on german population where authors have described the salvage rates and orchidectomy rates following exploration for torsion testes.***

***However, some points need clarification***

***Regarding the outcome measures that the authors have measured namely surgical infection rate , transfusion rate and length of hospital stay . how relevant are they with repsect to the procedure being described namely orchiectomy/ orchidopexy? how common is transfusion following orchidectomy? I believe transfusion following orchidectomy or orchidopexy is a very uncommon event unless the patient has associated comorbidities like CKD . the authors have further clarified in the beginning that the orchidectomy group had more comorbidities than the orchidopexy group which can explain the higher surgical infection rates ,transfusion rates and longer hospital stays .so when the authors are doing a regression analysis they should have accounted for these comorbidities too as they obviously have had a direct impact on the outcome variables measured .***

>>> We thank Reviewer 2 for the suggestions. The primary outcome of the present study was to assess the current trends in the management of suspected testicular torsion. As Reviewer 2 correctly states, perioperative complications after surgical exploration for suspected testicular torsion are extremely rare. More specifically, our analysis found surgical infection and transfusion rates below 1%. Accordingly, we performed a multivariable logistic and linear regression analysis to assess the perioperative outcomes (length of hospital stay, transfusion, and surgical wound infection) after surgical exploration due to suspected testicular torsion based on both the outcome of surgery (orchiectomy, detorsion with preservation of the testicle, and no testicular torsion) and based on the department of operation (urological versus non-urological). All models were adjusted for age, obesity, and diabetes. We could not adjust for further risk factors such as CKD due to the low surgical wound infection and transfusion rates (for example only: 14 and 52 transfusion cases after detorsion with preservation of testicle and orchiectomy, accordingly). Given that it is desirable to have a minimum of 10 events per variable included in the multivariable regression analysis, we could not add further risk factors to the multivariable regression analysis. Besides, it should be highlighted that a certain selection bias cannot be avoided, since there are inherent differences in the baseline characteristics of the included patients, for which we cannot account. The latter was added as a further limitation of the present analysis.

***Further , regarding salvage rates the authors have come to the conclusion that when surgery is done in a urology setup chances of salvage rates are higher , however with the data that was available to the authors ( where there is a dearth of patient related data ) this conclusion is difficult make as some of the important factors here is time sine symptoms which the authors don't have . what if the group of patients who came to urology department where a slightly better of group with less comorbidities and presenting early to the department while the other group which presented to non urology dept had more comorbidities and had a delayed diagnosis. so the conclusion that patients presenting in urology dept have better salvage rates is difficult to make in this present study .***

>>> We thank Reviewer 2 for the valuable comment. Indeed, even though urology departments were associated with better perioperative outcomes, no definite conclusions can be drawn and further studies in the field are mandatory. As reviewer 2 correctly states, based on the present analysis, it cannot be ruled out that patients who presented to urology departments had less comorbidities or presented earlier for treatment compared to non-urology departments. The latter introduces a certain selection bias to the present analysis. Based on the comments and the suggestions of Reviewer 2, the limitations were now expanded, and the conclusions were mitigated.

**Reviewer #4**

***Reviewer #4: A good documentation of the cases over a long period of time.***

***1. A significant number of pt (almost half of the documented cases) suspected and operated on the basis of symptoms and Doppler study showed no testicular torsion intraoperatively.***

***Among patients with no testicular torsion, torsion of the appendix testis was the cause of acute testicular pain in 9,861 (25%) cases and epididymitis in 3,161 (8%) cases.***

***What does your data on the preoperative Doppler study of all the patients in the study show in terms of diagnosis and does it correlate with the operative findings?***

>>> We thank Reviewer 4 for the question. Unfortunately, the present administrative data do not permit us to perform further analysis about preoperative Doppler study. Moreover, we cannot correlate the clinical with the operative findings of the included patients. This was added as a further limitation of the present analysis.

***2.What does this study suggest in terms of a) correct and timely diagnosis of testicular torsion and b) preventing unnecessary surgeries? Please include if any in your discussion.***

>>> We thank Reviewer 4 for the questions. Indeed, our findings indicate that a correct and timely diagnosis of testicular torsion is mandatory to save the testicle and prevent unnecessary surgeries. Based on the suggestion of Reviewer 4, an additional paragraph was added in the discussion section of our manuscript discussing this issue in detail.

***3.Your study shows better outcome in hands of urologists compared with non-urological fraternity. It is preferable to get the surgery done by urologist, however this type of surgery being an emergency should be able to be performed by general surgeon, pediatric surgeons when necessary. Better distribution and availability of urologist throughout the country and urologists guided training of non-urological fellows to perform with improved outcome whenever required.***

>>> We thank Reviewer 4 for the valuable comments. Although our study shows better perioperative outcomes in hands of urologists, it should be highlighted that testicular torsion is an absolute emergency. Therefore, general, and pediatric surgeons should be able to perform it when necessary. The latter was now added in the discussion section.
